# Measuring the impact of built environment factors on station-level contributions to link-level crowding using a novel crowding contribution index

**DOI:** 10.1038/s41598-025-27483-y

**Published:** 2025-12-10

**Authors:** Blessy David Xavier, Varun Varghese, Makoto Chikaraishi, Akimasa Fujiwara

**Affiliations:** 1 Graduate School of Advanced Science and Engineering, Hiroshima University, 1-3-2 Kagamiyama, Higashihiroshima, 739-8511 Hiroshima Japan; 2 Department of Humanities and Social Sciences, Indian Institute of Technology Tirupati, Chindepalle, 517619 Andhra Pradesh India

**Keywords:** Crowding contribution index (CCI), Built environment, Automated fare collection (AFC) data, Public transport, Transit demand management, Environmental impact, Civil engineering

## Abstract

Metro crowding undermines passenger comfort, operational efficiency and network reliability. While prior research has examined station-level and system-wide crowding, little attention has been given to quantifying how individual stations contribute to link-level overcrowding. This study addresses this gap by introducing the Crowding Contribution Index (CCI), a metric that quantifies the extent to which destination stations drive overcapacity flows on preceding links. The CCI is computed via a structured framework integrating Automated Fare Collection (AFC) and GTFS link-network data. Applied to over 80 million trips across 237 Delhi Metro stations, 142 200 hourly CCI values reveal that 46.35% of station-hours exceed capacity, with highest contributions clustered in specific stations. A Type II Tobit model assesses built-environment (BE) variables, showing that POI and intersection densities increase contributions, while POI entropy reduces them, underscoring land-use diversity’s role. Random Forest and XGBoost models corroborate these findings, ranking BE variables as the strongest CCI predictors. These insights emphasise the need for integrated land-use and transport strategies. The CCI framework offers operators a scalable tool for real-time service adjustments, such as targeted short-turns and dynamic fleet deployment, and guides planners toward sustainable, integrated land-use planning, making it especially valuable for rapidly urbanising, data-constrained cities.

## Introduction

Crowding in public transit systems is a critical concern that affects its attractiveness, operational efficiency, and system reliability^1^. There are multiple objective and subjective measures of crowding, which include passenger load factor (the ratio of passengers to vehicle capacity), seat occupancy rates, standee density, and crowd density per square metre. Objective measures, often derived from operational data, focus on quantifiable metrics like passenger counts or vehicle capacity utilisation. In contrast, subjective measures capture passenger perceptions of comfort and overcrowding, often gathered through surveys or observational studies^2^. While these metrics quantify “how full” vehicles are, they often obscure where and how congestion propagates through the network’s links, limiting planners’ ability to pinpoint congestion sources and design targeted interventions.

Technological advancements have further refined crowding measurements. Automated Fare Collection (AFC) systems, such as smart card data has transformed crowding analysis by recording individual tap-in/tap-out events at every station. Early AFC research focused on origin–destination (OD) estimation, using smart-card data to derive station-to-station flow matrices^3,4^. Building on this, Pelletier et al.^5^ outlined how AFC data informs strategic planning (long-term network expansions), tactical adjustments (schedule refinements), and operational monitoring (daily ridership and performance indicators). As AFC systems evolved to capture both entry and exit information, researchers developed schedule-based assignment methods, heuristics that match tap events to specific train trips by minimizing waiting times, lost time, transfers, and implausible itineraries^6–8^. These approaches enable estimation of segment-level passenger loads, thus refining our understanding of link-level crowding.

Integration with Automated Vehicle Location (AVL) data further refined crowding estimates. Cheriyamadam^9^ first combined AFC flows with AVL on the London Underground; subsequent studies by Zhu, Koutsopoulos & Wilson^10^, Hörcher, Graham & Anderson^11^, Yap, Cats & van Arem^12^, Basso et al.^13^, and Skoufas et al.^14^quantified standee density, time-weighted load factors, and crowding costs within route-choice models. AVL data provides the real-time location of trains, making it easier to match entry and exit information from AFC systems to specific trains and subsequently calculate various crowding indicators. However, accessing AVL data can be challenging, particularly in the context of the Global South, where even the integration of smartcards with transit systems is often limited. In such cases, other open data sources, such as General Transit Feed Specification (GTFS), can serve as valuable alternatives. GTFS data can be leveraged in combination with AFC data to estimate segment-level passenger loads, offering a practical solution in environments with limited technological infrastructure.

Building on these advances in AFC- and AVL-based crowding diagnostics, it is equally important to consider how the environments surrounding each station shape travel demand, and ultimately, in-vehicle congestion. Numerous studies have shown that station-area Built Environment (BE) characteristics such as land-use density, land-use diversity, street connectivity, and local economic vitality strongly influence metro ridership^3,4,15–18^. High densities of shopping, offices, schools, and healthcare facilities tend to generate more boarding and alighting activity, while well-connected street networks and mixed-use development promote transit-oriented travel patterns^19,20^. More recently, machine-learning based methods have uncovered that these BE effects are far from uniform: proximity to central business districts, parking availability, and even subtle variations in public-space amenities can all produce nonlinear ridership responses^21–24^. Multiple studies have used machine learning models with explainable capabilities to evaluate the impact BE variables^25,26^. Yet these contributions to station-level demand have rarely been linked directly to what happens inside the trains themselves. This disconnect stems from the challenge of reconciling two spatially distinct phenomena: station-level BE factors and crowding that occurs inside trains between stations.

In fact, a key gap persists, whereas built-environment research typically examines how land use drives boarding volumes, and crowding studies focus on in-vehicle loads, hardly any work traces those onboard counts back to the very station contexts that generate them. We lack a framework for asking, “Which station’s surroundings are most responsible for pushing link-level loads beyond capacity?” Without that connection, planners cannot evaluate whether zoning changes, pedestrian improvements, or mixed-use incentives will actually alleviate in-vehicle crowding or merely shift it elsewhere.

To bridge this gap, a novel indicator is needed to quantify each station’s contribution to link-level crowding. AFC data typically provides information about the origin and destination (OD) stations for each trip, which can theoretically be disaggregated into a series of links between consecutive stations. By aggregating data across all trips, it becomes possible to calculate how each destination station contributes to link-level crowding beyond a defined capacity threshold. Such an indicator would allow for a systematic analysis of the relationship between station-level BE factors and link-level crowding. Understanding this relationship would enable policymakers and planners to address congestion hotspots more effectively and optimise transit systems to balance ridership growth with passenger comfort and operational efficiency.

The study aims to address two key objectives. First, it proposes a novel Crowding Contribution Index (CCI) to quantify the role of destination stations in contributing to link-level crowding. This index captures the extent to which passengers alighting at a particular station contribute to overcapacity crowding on preceding transit links. Second, the study seeks to test the relationship between the CCI and station-level BE variables. By achieving these objectives, the study provides a comprehensive framework to understand how the BE influences crowding dynamics and offers actionable insights for improving transit planning and urban design. Unlike traditional metrics such as load factor or standee density that treat crowding as a train‑wide phenomenon, the CCI attributes excess passenger loads to the specific stations whose users generate them. Destination stations frequently coincide with high‑attraction land uses such as commercial hubs, business districts, educational campuses, that pull in large numbers of passengers. By tying in‑vehicle crowding back to these station areas, CCI would offer actionable insights: agencies can selectively increase service frequency or train capacity during peak periods at the stations that contribute most to overcrowding, and they can coordinate land‑use or demand‑management policies, such as targeted Transit-Oriented Development (TOD) incentives, to mitigate congestion and improve the overall passenger experience.

This study makes several significant academic contributions. First, it introduces a CCI that quantifies the role of destination stations in contributing to link-level crowding in metro rail systems. The CCI provides a new lens to systematically analyse crowding dynamics, bridging a critical gap in existing literature that rarely considers the impact of destination stations on link level crowding. Second, the study advances the understanding of the relationship between BE characteristics, guided by TOD principles, and link-level crowding. Finally, these contributions are applied to an empirical case study of the Delhi Metro Rail system, India’s largest and busiest rapid transit network. Delhi, as a rapidly growing megacity with a projected population of 30.9 million by 2041^27^, faces immense challenges in managing the increasing demand for public transit. The study leverages AFC data from September 2023, comprising 80,042,580 cleaned trips across 237 stations on the extensive Delhi Metro network. By applying the CCI, the research investigates how TOD-influenced BE characteristics around stations impact link-level crowding, providing unique insights into the dynamics of Delhi’s metro system. To date, only a handful of studies have leveraged AFC data for crowding analysis in Global South contexts. Empino et al.^28^ demonstrated how smart‑card records alone can predict daily ridership patterns on Manila’s metro system, while Pieroni et al.^29^ used AFC and machine‑learning methods to uncover how station‑area and operational factors drive bus‑corridor crowding in São Paulo. Unlike these demand‑forecasting efforts, our CCI framework uniquely decomposes in‑vehicle overcrowding by allocating excess link‑level passenger loads directly to the destination stations whose BE characteristics drive them, providing the first station‑level linkage between urban form and link level crowding.

The remainder of this paper is structured as follows. Section Methods describes the data sources, preprocessing steps, and methodology for computing the CCI and BE variables, along with the specification of our Type II Tobit and machine‑learning models. Section Results presents the empirical results, including Tobit estimates and out‑of‑sample performance of Random Forest and XGBoost algorithms, supplemented by SHAP-based feature importance analyses. Section Conclusions concludes by summarizing the key findings, discussing policy implications and practical recommendations, acknowledging limitations, and suggesting directions for future research in integrated crowding analytics and urban planning.

## Methods

### Study area: Delhi metro system

This study evaluates the impact of BE variables on crowding and congestion within the Delhi Metro system. With an estimated population exceeding 30 million and an area of 1,486.5 km²^30^, Delhi faces significant urban mobility challenges. The Delhi Metro, operational since 2002, addresses these challenges with a network spanning 392.44 km and comprising 288 stations, including those operated by the Noida Metro Rail Corporation (NMRC) and the Airport Express Line, managed by Delhi Airport Metro Express Pvt. Limited (DAMEL). The metro network also serves the adjoining cities in the National Capital Region (NCR), such as Gurugram, Noida, Faridabad, Ghaziabad, and Bahadurgarh.

**Fig. 1:**
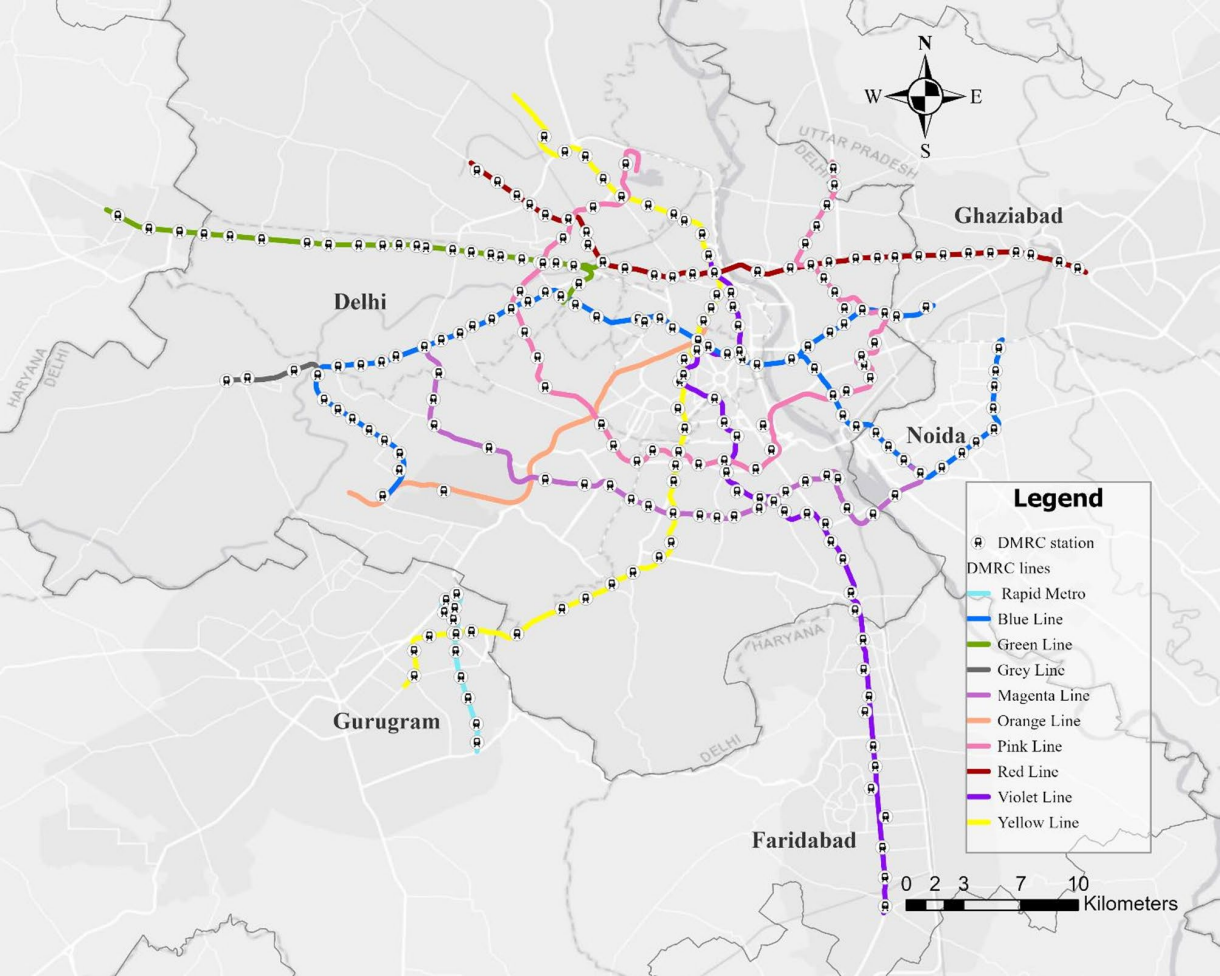
Study area and network map of the Delhi Metro system. (The figure was created using ArcGIS Pro version 3.1.0 (Esri Inc., https://www.esri.com)). The basemap sources include Esri, DeLorme, HERE, and MapmyIndia.

This study uses data from 237 stations managed by the Delhi Metro Rail Corporation (DMRC). The dataset includes tap-in and tap-out ticketing records from the Yellow, Violet, Red, Magenta, Grey, Green, Blue, and Orange (Airport Express Line) lines, as well as the Rapid Metro Line connecting Gurugram to Delhi (see Fig. 1). Common junction stations serving multiple lines were considered only once to avoid duplication. To analyse the influence of BE factors on metro crowding, this study integrates AFC data from smart cards and QR codes with Point of Interest (POI) data and Meta’s Relative Wealth Index (RWI) data^31^. By combining these datasets, the research aims to uncover how the built environment contributes to crowding and to identify strategies for efficient crowd management within the metro network.

### Automated fare collection data

The smartcard and QR code data for this study were collected from the AFC system of DMRC. In Delhi, passenger data is primarily collected through smartcards, which are reusable, and QR codes, which act as tokens for single trips. This dataset spans a 30-day period, from September 1, 2023, to September 30, 2023, and includes data from 237 metro stations. During this timeframe, a total of 80,398,750 trips were recorded. Each trip is associated with a unique ID (physical ID), containing details such as entry and exit station IDs, tap-in (datetime of entry) and tap-out (datetime of exit) timestamps in the YYYY-MM-DD HH: MM: SS format, and the fare deducted.

**Fig. 2:**
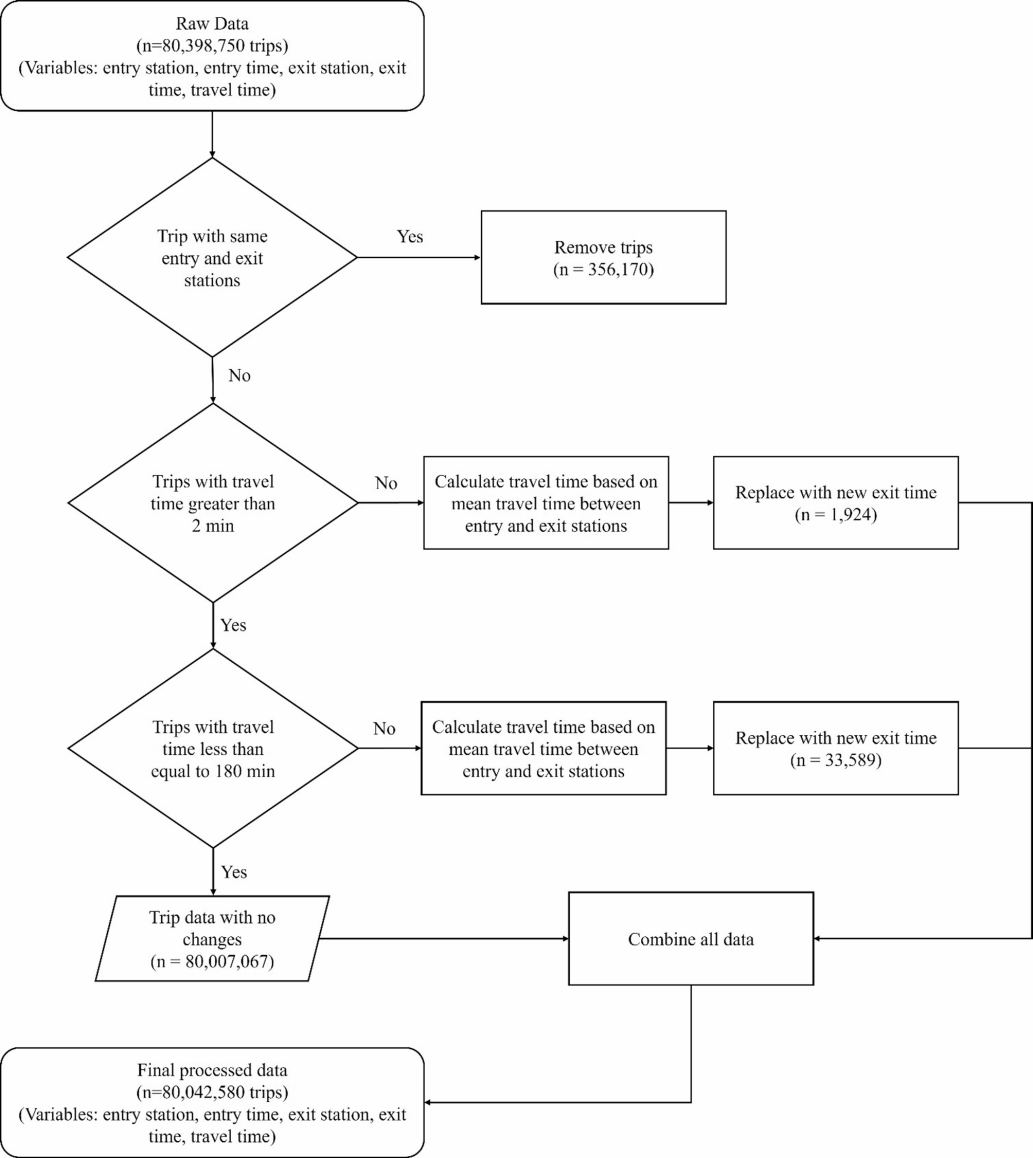
Data preprocessing workflow for AFC system records of the Delhi Metro.

To ensure accuracy, trips where the entry and exit stations were the same were identified as invalid and removed, resulting in the exclusion of 356,170 trips (0.44% of the total dataset). Anomalies in travel time were addressed by recalculating travel times based on the mean travel time between the respective entry and exit station pairs. Specifically, trips with a travel time of less than or equal to 2 min (1,924 trips, 0.002%) or greater than 180 min (33,589 trips, 0.04%) were corrected with updated datetime of exit values. The threshold of 2 min was chosen as travel times below this duration are assumed to be infeasible for valid passenger movement between stations. Similarly, a travel time exceeding 180 min were assumed to be unrealistically large given the extent of Delhi’s metro network and was therefore adjusted. Such data-cleaning steps are common in AFC-based analyses^32,33^ and, since they affect only a tiny fraction of all records, are unlikely to materially impact our results. Valid trips with no anomalies, accounting for 80,007,067 trips (99.51% of the dataset), were retained without modifications. After combining all processed data, the final dataset comprised 80,042,580 trips, including key variables such as physical ID, entry station, exit station, entry time, exit time, and travel time. The details of this preprocessing are illustrated in Fig. 2.

### Built environment data

The categorisation of PoIs includes 12 distinct types, as outlined in Table 1, which also provides detailed descriptions and subcategories for each type. The data, sourced from the National Capital Region Planning Board (NCRPB) and OpenStreetMap (OSM), underwent a deduplication process to consolidate overlapping categories. Multiple subcategories with shared characteristics were grouped into the 12 categories presented in Table 1. These PoIs were collected within a 1200-metre radius of metro stations, selected to capture the immediate urban catchment area influenced by metro accessibility^34^. This radius was chosen to ensure the inclusion of key amenities and services supporting transit-oriented development. While studies such as Chen et al.^35^used a 500-metre radius for PoIs, a larger radius was considered for Delhi to account for places accessible not only by walking but also by paratransit feeder modes such as autorickshaws. The PoIs were extracted from the data sources in July 2024. While any aggregation of dozens of raw OSM/NCRPB classes into 12 umbrella types necessarily requires judgment, our scheme closely follows the native OSM feature hierarchy and the NCRPB land-use taxonomy, grouping only those subcategories with clear functional affinity (e.g. cinemas, restaurants and theme parks all under “Leisure,” schools and colleges under “Education,” etc.). To maximize transparency and reproducibility, the full mapping from original subcategories to our 12 types is provided in Table 1.

**Table 1:** Categorisation of points of interest (PoIs).

Type of PoI	Description	Mean (std. dev)	Source
Banking	Banks, ATMs	112.22 (68.35)	NCRPB, OSM
Bus stops	Bus stops	12.16 (12.46)	OSM
Education	Schools, Colleges	29.11 (22.52)	NCRPB, OSM
Health	Hospitals, Pharmacy, Pharmaceutical labs, clinics (including veterinary)	16.97 (10.94)	NCRPB, OSM
Hotels	Hotels, Lodging	4.05 (12.32)	NCRPB, OSM
Industry	Inland container depots, Industrial estates, Technology parks, Factories, Other industrial areas	0.56 (0.75)	NCRPB
Leisure	Heritage and tourism, Sports facilities, Cinemas, Golf course, Fountains, Restaurants, Theme parks, Other leisure points	36.85 (43.71)	NCRPB, OSM
LIG housing	Slums, Slum redevelopment colonies	3.22 (7.07)	NCRPB
Open space	Parks, National parks, Bird Sanctuaries, Playgrounds	32.41 (34.21)	NCRPB, OSM
Parking	Parking	0.65 (1.6)	NCRPB
Public utility	Dams, Post offices, Warehouses, Fire stations, Fuel stations, Library, Police stations, Rain gauge stations, Sewage treatment plants, Toilets, Laundry, Underground reservoirs, Public offices, Other utility services	45.07 (49.44)	NCRPB, OSM
Shopping	Supermarkets, Weekly markets, shopping malls, Shops, Other shops and markets	21.19 (34.39)	NCRPB, OSM

**Fig. 3:**
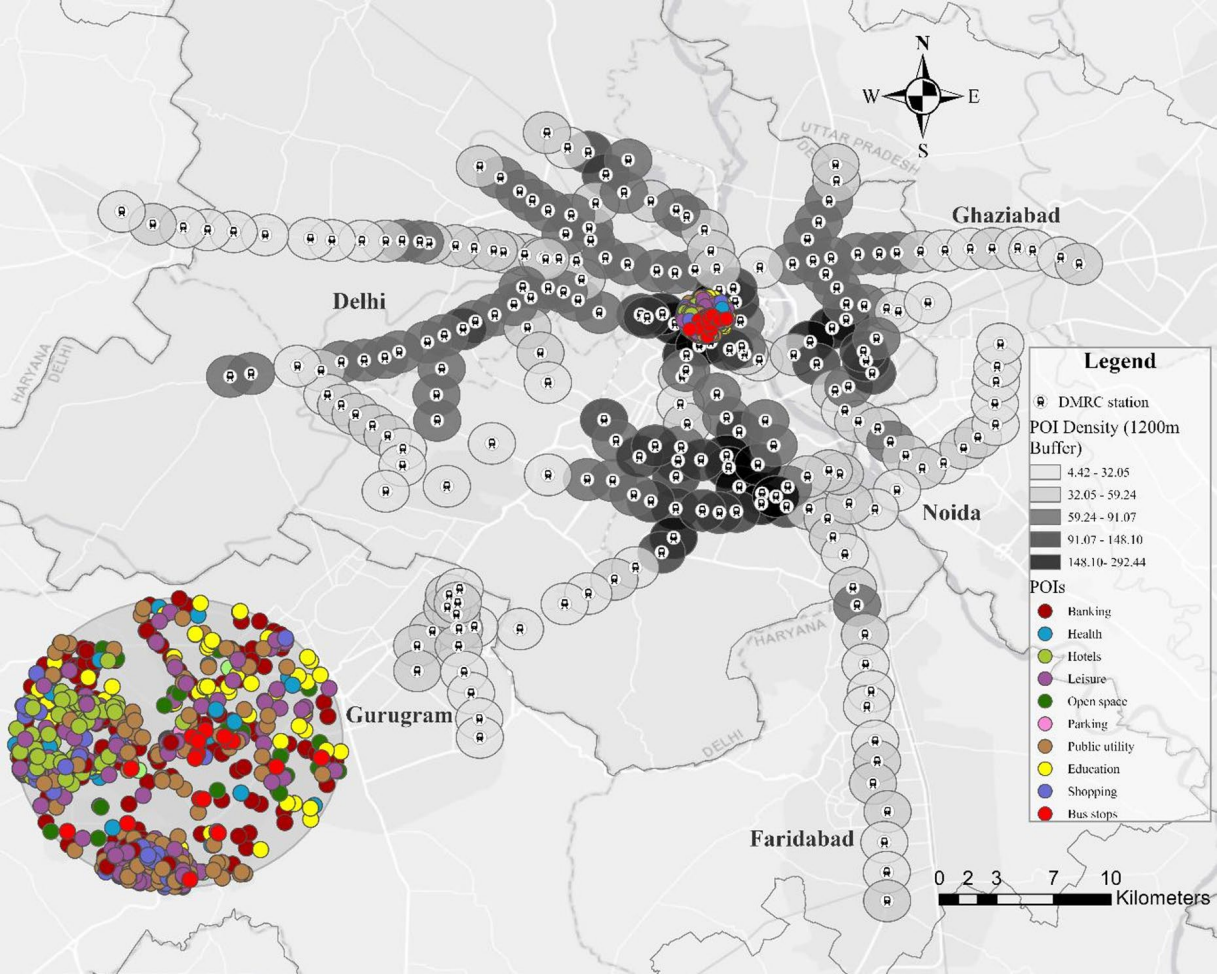
Points of Interest (PoI) categories and density across metro stations in Delhi. (The figure was created using ArcGIS Pro version 3.1.0 (Esri Inc., https://www.esri.com)). The basemap sources include Esri, DeLorme, HERE, and MapmyIndia.

The dataset encompasses a total of 74,530 PoIs within the 1200 m buffer of 237 stations, distributed across categories such as Banking (26,596), Health (4,023), Hotels (960), Industry (132), LIG Housing (763), Leisure (8,734), Open Space (7,681), Public Utility (10,681), Shopping (5,023), Parking (155), Education (6,900), and Bus Stops (2,882). This categorisation provides a structured view of urban functionality, supporting comparative analysis of metro station surroundings. Figure 3 shows the distribution of PoI density and categories across Delhi. However, it can be observed that if only NCRPB data were available, the PoI counts would be significantly smaller due to its limited coverage. Nonetheless, it is assumed that such missing data is randomly distributed across stations, making the dataset suitable for comparative analysis. Additionally, certain PoIs, such as shopping malls, could fit into multiple categories like Shopping and Leisure; however, for simplicity, they have been assigned to a single category. Other limitations include potential inaccuracies in the data sources and variability in the granularity of mapped PoIs, which might affect the comprehensiveness of the analysis.

The density considered in this study represents the total number of PoIs per unit area within the buffer zone (in km^2^). Meanwhile, entropy is calculated using the Shannon entropy formula and is represented as:

1 $$\:{H}_{s}=-\sum\:_{i=1}^{n}{p}_{is}ln{p}_{is}$$

where,

$\:{H}_{s}$= Shannon entropy for station $\:s$,

$\:n$= number of PoI categories with nonzero counts,

$\:{p}_{is}$= proportion of PoIs in category $\:i$ at station $\:s$, calculated as:

2 $$\:{p}_{is}=\frac{{x}_{is}}{{\sum\:}_{j=1}^{n}{x}_{js}}$$

where, $\:{x}_{is}$ is the count of PoIs in category $\:i$ for station $\:s$. For this study, PoIs were classified into 12 categories ($\:i$), including Banking, Health, Hotels, Industry, LIG Housing, Leisure, Open Space, Public Utility, Shopping, Parking, Education, and Bus Stops.

Apart from PoI data, we utilised Meta’s RWI data, which provides a standardised measure of regional wealth based on indicators such as digital ad engagement, device usage, and higher education proportion^31^. The RWI data is used as a proxy for economic indicators around the station, as it is well established that wealthier regions often have better infrastructure and services, leading to higher transit demand^36^. Additionally, we considered intersection density, which reflects the degree of street connectivity around each station. Higher intersection density typically indicates a more walkable environment, which can facilitate better access to transit and influence ridership patterns.

**Fig. 4:**
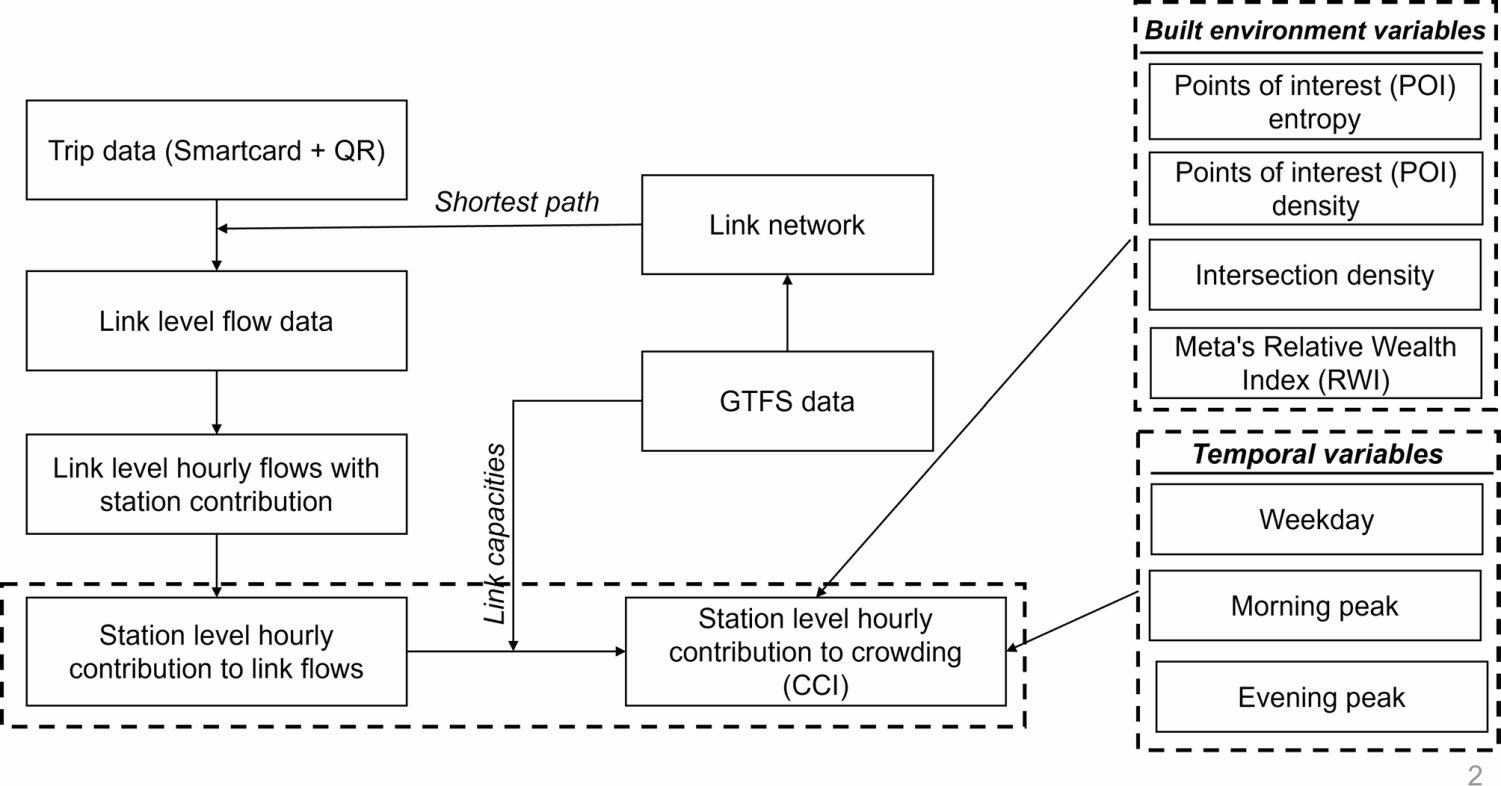
Methodological framework adopted in the study.

The primary objectives of this paper are to explore the relationships between BE variables and the contribution of each station to link-level crowding. Figure 4 presents the methodological framework adopted in the paper. This framework includes the calculation of link-level flows using trip data from AFC and the link network derived from GTFS data. These link-level flows are then used to calculate the hourly station-level contributions, i.e., the CCI. Finally, a statistical model is estimated to evaluate the impact of BE variables on CCI. Additionally, the impact of time of day (peak vs. non-peak hours) and day of the week (weekday vs. weekend) was also evaluated. This section elaborates on the methodologies adopted in this study.

### Link level flow

The cleaned AFC dataset comprised 80,398,750 trips, capturing detailed entry and exit information, including timestamps and station identifiers. This data was integrated with GTFS data to construct a transit network graph using the igraph package in R^37^. GTFS data provided essential structural details by linking stops, routes, and trips through sequence data and travel times. Stop times were processed to calculate travel times between consecutive stops, which were then used to create weighted edges representing connections within the network. These weights, based on average travel times enabled the evaluation of the most efficient transit paths between stations. The resulting directed network facilitated the calculation of shortest paths for each pair of entry and exit stations.

A shortest path function, based on Dijkstra’s algorithm, was used to compute the path between entry and exit stations by considering the weights assigned to each link. The total travel time, calculated as the difference between the entry and exit times, was distributed across the links proportionally to their respective weights. It is important to note that this process does not account for the time travellers spend walking from the AFC gate to the train at the entry station or from the train to the gate at the exit station, which is a limitation of the adopted method. We recognise that distributing trips via a deterministic shortest-path (Dijkstra) assignment neglects route-choice variability, intermediate transfers, and time-dependent effects such as train bunching or platform queuing. However, on the high-frequency, predominantly radial Delhi Metro network, most passengers naturally follow minimal-time itineraries and alternative routings are limited. Using GTFS-derived link weights and a shortest-path algorithm thus provides a pragmatic, computationally efficient first–order approximation for very large AFC datasets to link-level flows. Each trip is then represented as a series of links, capturing details such as start and end nodes, travel times, and associated temporal information (entry and exit times). The process generated 1,129,220,894 individual links, representing detailed connections within the transit network. The resulting output is a comprehensive link-level dataset, enabling a granular evaluation of transit performance and user behaviour. Future work will extend this with probabilistic assignment models and explicit treatment of transfer and walking times.

### Crowding contribution index (CCI)

The generated link-level information is then utilised to create an hourly link-level flow dataset. This dataset consolidates the link-level flows for each hour, day, and link ($\:{F}_{lhd})$. Additionally, the dataset calculates how this hourly link-level flow is distributed across each station $\:\left(s\right)$ based on their destination locations. Subsequently, the flow ratio $\:{(r}_{slhd})$ is calculated, which determines the proportional contribution of a station’s flow to the total flow on a link during an hour on each day. For each station and link, the ratio is calculated as the flow on each link with the destination station $\:s$ divided by the total flow on the link, but only if the total flow exceeds the link’s capacity ($\:{C}_{lhd}$) during the given hour and day. The link capacity is derived from GTFS data, assuming 200 people per car and 6 cars per train. This assumption is considered reasonable, as also identified by the DMRC^38^. If the total flow does not exceed the capacity, the station’s contribution is set to zero. This ensures that the calculation focuses on crowding conditions where capacity constraints are exceeded, capturing the relative impact of each station on link-level crowding.

3 $$\:{r}_{slhd}=\left\{\begin{array}{c}\frac{{f}_{slhd}}{{F}_{lhd}},\:if\:\:{F}_{lhd}>{C}_{lhd}\\\:0,\:\:Otherwise\end{array}\right.$$

Where,

$\:{f}_{sl}$ = Flow on link $\:l$ with destination at station $\:s$ during hour $\:h$ on day $\:d$.

$\:{F}_{lhd}$ = Total flow on link $\:l$ during hour $\:h$ on day $\:d$.

$\:{C}_{lhd}$ = Capacity of the link $\:l$ during hour $\:h$ on day $\:d$.

$\:{r}_{slhd}$ = Ratio of the flow with destination station $\:s$ to the total flow on link $\:l$ during hour $\:h$ on day $\:d$.

**Fig. 5:**
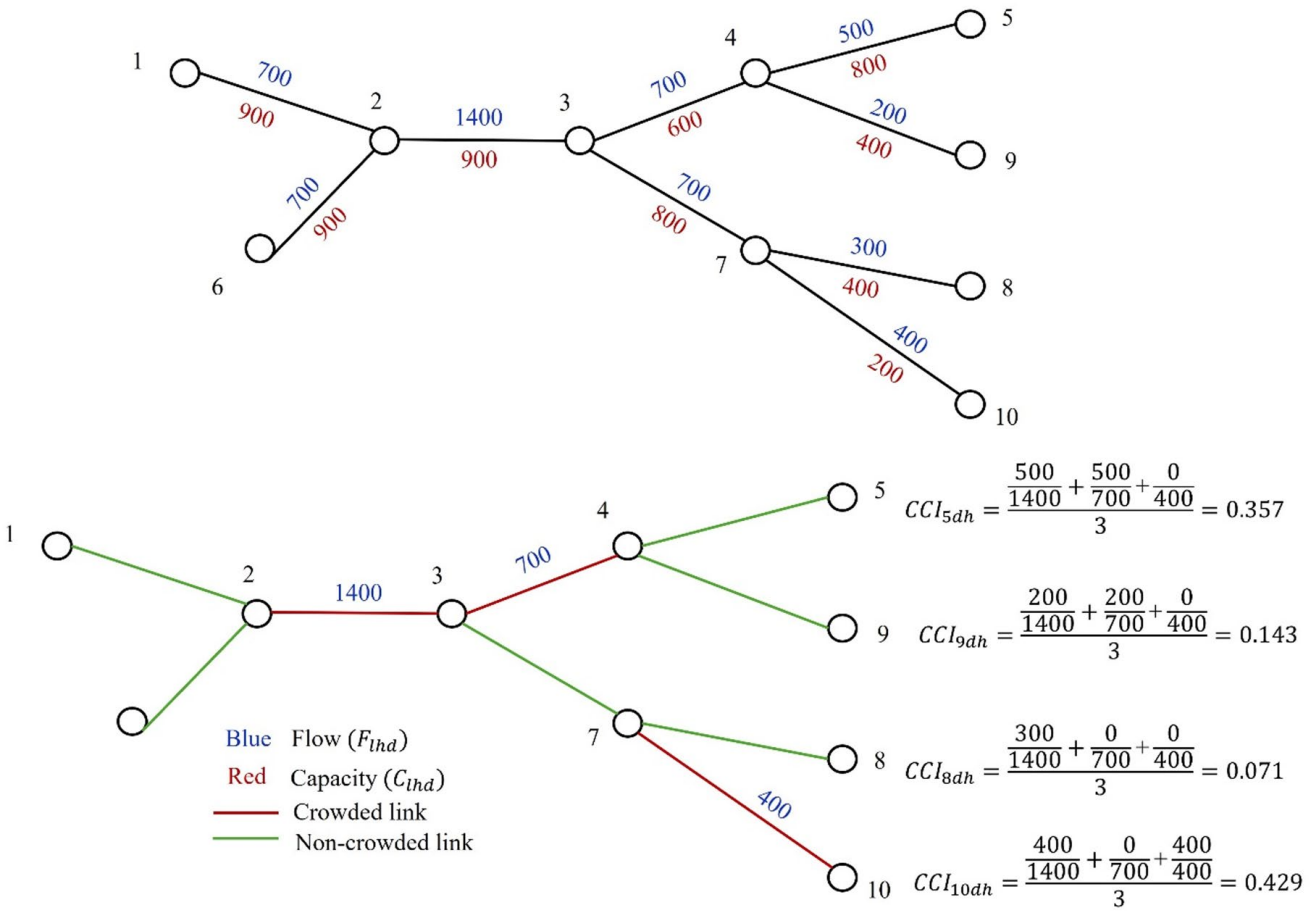
Illustration of the process for calculating the Crowding Contribution Index (CCI).

Crowding contribution index for a station $\:{(CCI}_{shd})$, represents the average contribution of all link flows directed toward a specific destination station $\:s$ during a given hour $\:h$ on a given day $\:d$. It is calculated as the mean of the flow ratios $\:{(r}_{slhd})$ for all links that contribute to the destination station $\:s$, where the total flow on each link exceeds it capacity. Equations (3), (4), and Fig. 5 illustrate the calculation process for $\:{CCI}_{shd}$. By aggregating the flow ratios of links directed toward $\:s$, the $\:{CCI}_{shd}$ quantifies the degree to which these stations contribute to potential crowding at the link-level during specific time intervals.

4 $$\:{CCI}_{shd}=\frac{1}{\left|{L}_{hd}\right|}\sum\:_{l\in\:{L}_{hd}}{r}_{slhd}$$

where,

$\:{CCI}_{shd}$= Crowding contribution index of station $\:s$ during hour $\:h$ on day $\:d$.

$\:{L}_{hd}$= Set of links during hour $\:h$ on day $\:d$ where $\:{r}_{slhd}\ne\:0$.

$\:\left|{L}_{hd}\right|$= Number of links with non-zero contributions for station $\:s$ during hour $\:h$ on day $\:d$.

While this study primarily examines the role of destination stations in contributing to link-level crowding, origin stations also play a significant role in shaping congestion patterns. Although crowding physically accumulates from boarding events, attributing link-level overcapacity flows to destination stations allows us to tie crowding back to the built-environment around trip ends. In other words, by measuring “which station’s draw” pushes a link beyond capacity, the CCI becomes an explanatory lens for how BE factors drive crowding dynamics, rather than a literal account of where passengers board. This station-level attribution offers planners actionable targets (e.g. where to add rolling stock or adjust headways) even if it abstracts away detailed load-propagation mechanics. Additionally, this study defines overcapacity crowding using a threshold based on capacity ($\:{C}_{lhd})$. This threshold can be adjusted if policymakers are interested in identifying only extreme overcrowding. In such cases, it could be set to twice the capacity or another suitable value to reflect more severe congestion conditions.

A major limitation of this work was that we lack direct AVL or on-board load measurements for the Delhi Metro. Instead, we derive link capacities from GTFS (200 pax/car, 6 cars/train, per DMRC guidelines) and focus on passengers exceeding those thresholds. By framing CCI as a relative measure, i.e. the share of “overcapacity” flow attributed to each destination station, we mitigate systematic biases in absolute load estimates. While AVL-based validation would strengthen empirical credibility, the CCI’s comparative nature ensures robust station-level insights even when only AFC and GTFS data are available. Crucially, this CCI methodology is designed as a flexible framework, it can be readily extended to incorporate probabilistic transit assignment, intermediate transfers, and AVL-derived occupancy data in future studies.

### Regression analysis

To investigate the relationship between BE factors and CCI, we employ a comparative regression framework that compares classical econometric techniques with Machine Learning (ML) methods. This approach addresses limitations associated with traditional parametric models in capturing complex, nonlinear interactions and potential spatial dependencies associated with BE factors influencing public transport crowding. Initially, a Type II Tobit (sample selection) model^39^ is utilised due to its ability to correct for potential selection bias arising from observing CCI only for those station-hour instances contributing to crowded links. This method involves jointly estimating a Probit selection equation indicating whether a station contributes to crowding, and an outcome equation predicting CCI conditional upon selection. The model is estimated using a Maximum Likelihood Estimation (MLE) approach, jointly estimating the selection and outcome equations.

To capture more complex, potentially nonlinear interactions among variables, we also incorporate multiple ML algorithms into our analysis: Ordinary Least Squares (OLS) regression, serving as a baseline linear benchmark; Ridge and Lasso regression methods, which introduce L2 and L1 regularisation, respectively, for handling multicollinearity and enabling feature selection^40^; Random Forest, an ensemble decision-tree method known for robust nonlinear modelling capabilities^41^; and Extreme Gradient Boosting (XGBoost), a gradient-boosting framework noted for its high predictive performance and effectiveness in modelling intricate data structures^42^. Such ML approaches have shown promise in recent urban and transportation studies, particularly in capturing nonlinearities associated with travel demand and behavioural patterns^43,44^.

The dataset was randomly divided into training (80%) and testing (20%) sets. All regression and ML models were trained using the training subset, with out-of-sample predictive performance evaluated using the held-out test set. Hyperparameter tuning for ML algorithms was conducted through three-fold cross-validation on the training data, selecting parameter combinations that minimised the cross-validated mean squared error (MSE). The tuned hyperparameters and their corresponding candidate values are summarised in Table 2.

**Table 2:** Hyperparameters tuned for ML algorithms.

Model	Hyperparameters	Values Tested
Ridge Regression		[0.01, 0.1, 1, 10, 100]
Lasso Regression		[0.001, 0.01, 0.1, 1, 10]
Random Forest	Number of estimators	[100, 150, 200, 250, 300]
	Maximum tree depth	[10, 20, 30]
	Minimum samples split	[2, 5]
	Minimum samples per leaf	[1, 2]
XGBoost	Number of boosting rounds	[100, 150, 200]
	Learning rate	[0.05, 0.1, 0.2]
	Maximum tree depth	[4, 6, 8]
	Subsample ratio	[0.8, 1]
	Feature subsample ratio	[0.8, 1]

Table 3 provides a concise overview of all variables included in our analysis, spanning the dependent CCI metric, built-environment attributes, and temporal variables. Additionally, it is to be noted that CCI is handled differently across methods: in the Tobit framework it is split into a binary selection and a continuous outcome component, while in the machine-learning models it is used directly as a continuous regression target.

**Table 3:** Variables used in the type II Tobit and ML models.

Category	Variable	Description
Dependent Variable	Crowding Contribution Index (CCI)	Continuous share of link-level flow exceeding capacity, aggregated at station level; in Tobit models it is split into a binary selection equation (CCI > 0) and a continuous outcome equation (magnitude of CCI), whereas in all ML models it enters directly as the continuous regression target.
Built-Environment	PoI density	Total number of points of interest per km² within a 1,200 m buffer of each station.
	PoI entropy	Shannon entropy of the distribution of PoI categories within the station buffer (land-use diversity)
	Relative Wealth Index (RWI)	Proxy for local socioeconomic activity around each station (Meta’s RWI)
	Intersection density	Number of street intersections per km² within the station buffer (walkability/ connectivity).
Temporal variables	Weekday dummy	1 = Monday–Friday; 0 = Saturday–Sunday.
	Morning peak dummy	1 = 07:00–10:00 AM; 0 otherwise.
	Evening peak dummy	1 = 05:00–08:00 PM; 0 otherwise.

## Results

### Crowding contribution index (CCI) analysis

**Fig. 6:**
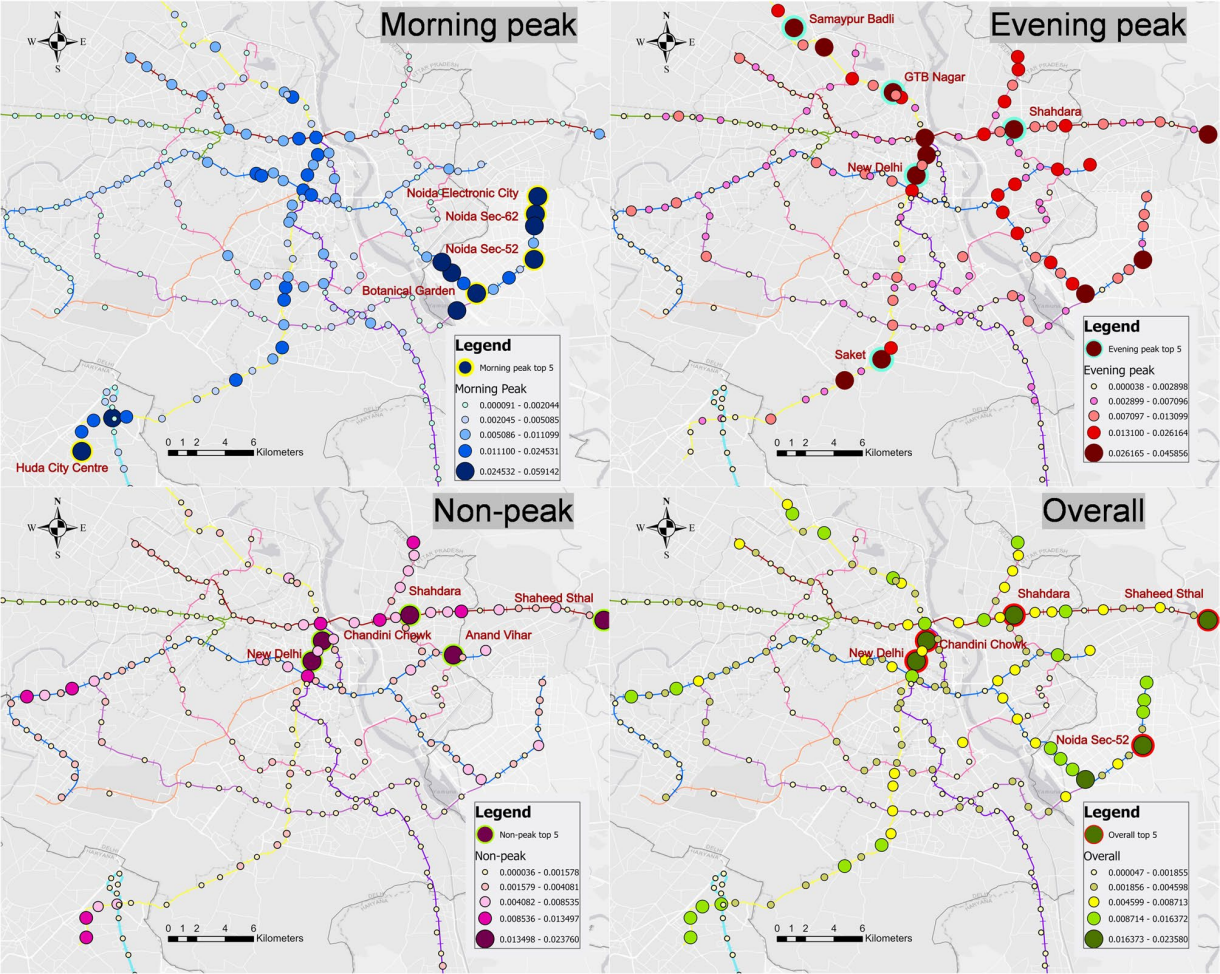
Spatial distribution of metro stations with the highest CCI values. (The figure was created using ArcGIS Pro version 3.1.0 (Esri Inc., https://www.esri.com)). The basemap sources include Esri, DeLorme, HERE, and MapmyIndia.

A descriptive analysis of CCI values highlights the metro stations in Delhi that contribute most significantly to link-level crowding. CCI was calculated at every hour for each of the 237 stations, resulting into a total of 142,200 CCI values, of which 76,289 (53.65%) were zero, indicating that no station contributed to flows over capacity in any of the links. Of the remaining 65,911 (46.35%), the patterns of crowding vary across the network, with the highest CCI values (top five) concentrated in specific time clusters (see Fig. 6).

During the morning peak (07:00–10:00), the five highest destination CCI values are at Noida Sector 62 (CCI ≈ 0.0591), Noida Sector 52 (CCI ≈ 0.0537), Botanical Garden (CCI ≈ 0.0496), Huda City Centre (CCI ≈ 0.0477) and Noida Electronic City (CCI ≈ 0.0411) (see Fig. 6). All except Huda City Centre lie on the eastern extensions into Noida, where large office parks, IT campuses and industrial estates draw thousands of commuters each morning. Botanical Garden metro station is the only interchange in Noida between the Blue and Magenta Lines, so it receives heavy alightings from both branches. Huda City Centre, at the southern end of the Yellow Line in Gurgaon’s corporate district, also records a high CCI as workers arrive for office and commercial activity. These concentrated arrivals at major employment hubs outpace the capacity set by train frequency and size, causing the links feeding into these stations to exceed their design limits. Additionally, the distribution across the entire network confirms a tightly clustered morning peak pattern, with elevated CCIs largely confined to Noida and Gurgaon regions, indicating a strong pull into a limited set of work-centre destinations.

During the evening peak (17:00 to 20:00), the highest destination CCI values shift to stations embedded in residential and mixed-use districts across the network (see Fig. 6). GTB Nagar (CCI ≈ 0.0449) on the Yellow Line’s northeast corridor lies beside dense housing estates and small marketplaces; here, large numbers of commuters alight to return home, exceeding the carrying capacity. Samaypur Badli (CCI ≈ 0.0391), the Red Line’s northern terminus, serves established residential colonies and local *bazaars*, generating similarly heavy evening alightings. Further east, Shahdara (CCI ≈ 0.0422) on the Red Line extension draws travellers from both residential and light-industrial areas, producing sustained upstream crowding. New Delhi station (CCI ≈ 0.0400), located beneath the city’s central business district and intercity rail complex, ranks among the top evening destinations as office workers, hospitality staff and transfer passengers disembark for hotels, restaurants and intercity trains. In South Delhi, Saket (CCI ≈ 0.0458), set within a mixed-use precinct of residential complexes, shopping malls, offices and cinemas, also emerges as a key evening destination, with leisure and return-home trips pushing upstream links beyond capacity. Meanwhile, the distribution across network shows the evening peak to be more spatially spread out than the morning showing elevated CCIs appear along multiple corridors, consistent with homebound flows dispersing toward many neighbourhoods rather than concentrating on a few central work hubs.

During non-peak hours (10:00–17:00 and after 20:00), several of the same stations that dominate overall CCI also rise to the top: New Delhi (CCI ≈ 0.0168), Chandni Chowk (≈ 0.0207), Shahdara (≈ 0.0237), Shaheed Sthal (≈ 0.0238) and, among others, Anand Vihar (≈ 0.0165). When we average CCI overall operating hours, New Delhi (≈ 0.0230), Chandni Chowk (≈ 0.0228), Shahdara (≈ 0.0237), Shaheed Sthal (≈ 0.0217) and Noida Sector 52 (≈ 0.0236) remain the five highest contributors. What these stations share is a strong connection to other transport modes. New Delhi and Chandni Chowk link to regional rail and major bus terminals, Shaheed Sthal sit beside large bus depots, and Anand Vihar is an intermodal hub for long-distance coaches. Additionally, these stations have a highly diverse mix of land uses (high PoI entropy) including offices, markets, and leisure. That combination of transfer function and mixed-use catchment generates steady alightings that exceed what the less-frequent off-peak service can carry.

**Fig. 7:**
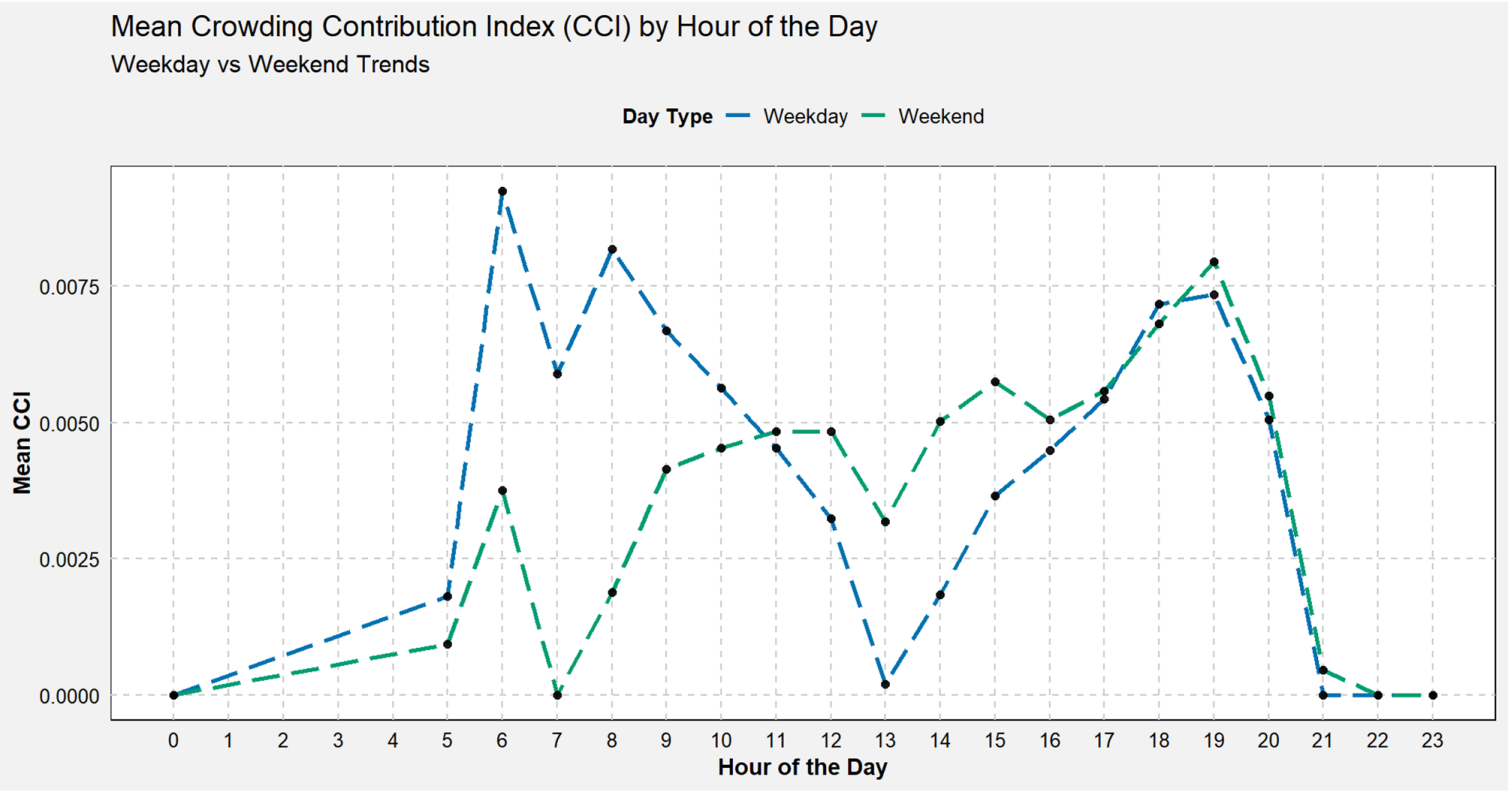
Mean CCI by hour of the day and day of the week.

Hourly CCI variations on weekdays show a rapid rise at 6 AM as early-shift commuters begin their journeys, followed by a slight dip at 7 AM despite continued high ridership. That dip occurs because the metro increases train frequency at the official start of the morning peak, temporarily boosting capacity enough to keep flows within design limits (CCI remains low whenever volumes do not exceed capacity). By 8 AM, passenger arrivals intensify beyond even the increased capacity and CCI reaches its highest morning peak values. After 10 AM, CCI falls to its lowest around 1 PM as commuter flows subside and midday service remains steady. A modest uptick between 2 PM and 3 PM likely reflects discretionary trips to shopping, education or appointments. The clear evening peak between 5 PM and 8 PM then appears as homeward and leisure travel again pushes arrivals above what the preceding service can carry (see Fig. 7).

Weekend patterns differ. There is no sharp early-morning jump, CCI remains comparatively low until early afternoon, when leisure and shopping trips build and then a 7 PM peak roughly matches weekday evenings. Overall, weekdays show pronounced peaks with a temporary 7 AM capacity buffer, while weekends exhibit a more even distribution of crowding contributions across the day.

### Determinants of CCI

The Type II Tobit (sample selection) model results indicate that several BE variables significantly influence whether a metro station contributes to crowding (see Table 3). PoI density exhibits a statistically significant positive effect, suggesting that stations surrounded by higher concentrations of shopping centres, educational institutions, healthcare facilities, leisure destinations, and other urban amenities are more likely to contribute to link-level crowding. Intersection density also has a positive and significant effect, implying that metro stations embedded within well-connected street networks are more prone to act as sources of crowding. Temporal factors such as weekday travel, as well as morning and evening peak periods, further increase the likelihood of a station’s contribution to crowded links. In contrast, PoI entropy and Meta’s RWI do not have significant effects on the probability of contributing to crowding.

The outcome equation of the Tobit model reinforces the importance of PoI density, which remains a strong positive predictor of CCI intensity. Stations with a greater number of surrounding points of interest experience higher crowding at the link level. PoI entropy displays a significant negative effect, indicating that a more balanced and diverse mix of land uses helps to distribute passenger flows and can reduce severe crowding at individual stations. Meta’s RWI, a proxy for local economic activity is positively associated with CCI in the outcome equation. Intersection density and weekday remain positive predictors, while both morning and evening peak indicators are negative, possibly reflecting a relative normalization of crowding during these times due to widespread system congestion. In contrast, during off-peak periods, crowding may be more concentrated at specific stations due to factors such as reduced service frequencies, event-driven demand surges, or localized land-use characteristics that generate disproportionately high passenger volumes. This difference in impact of hour of the day between the selection and outcome equations underscores the complex relationship between temporal demand and the spatial distribution of congestion. While peak hours drive higher ridership system-wide, the intensity of each station’s contribution to link-level crowding may be mitigated by operational factors such as train capacity, headway adjustments, and demand-spreading strategies.

The estimated Rho parameter is not statistically significant, implying that unobserved factors influencing whether a station contributes to crowding do not strongly affect the extent to which it does so. This suggests that selection bias correction is not a major concern in this model. Meanwhile, the estimate of the standard deviation of the error term indicates the variability in the intensity of CCI. The results suggests that there is substantial unexplained variation in crowding intensity across stations, even after accounting for observed factors. Additionally, the out-of-sample R² for the Tobit outcome equation is 0.025, indicating limited predictive power when compared to nonlinear ML models.

**Table 4:** Estimation results of the type II Tobit model for CCI.

Variable	Estimate	Std. Error	t value
Selection			
Intercept	***-0.885***	***0.0393***	***-22.528***
PoI density	***0.00045***	***0.00010***	***4.494***
PoI entropy	-0.0046	0.0191	-0.243
RWI	0.0159	0.0174	0.913
Intersection density	*0.0047*	*0.0019*	*2.397*
Weekday	***0.191***	***0.0090***	***21.371***
Morning peak	***1.248***	***0.0103***	***121.355***
Evening peak	***1.879***	***0.0121***	***155.875***
**Outcome**			
Intercept	***0.0153***	***0.00083***	***18.563***
PoI density	***4.54 × 10⁻⁵***	***1.89 × 10⁻⁶***	***24.031***
PoI entropy	***-0.0073***	***0.00036***	***-20.432***
RWI	***0.0031***	***0.00033***	***9.636***
Intersection density	***0.00016***	***3.65 × 10⁻⁵***	***4.353***
Weekday	*0.00042*	*0.00018*	*2.334*
Morning peak	*-0.0028*	*0.00029*	*-9.798*
Evening peak	*-0.0033*	*0.00034*	*-9.709*
Sigma ($\:\sigma\:)$	*0.0180*	*5.55 × 10⁻⁵*	*324.33*
Rho ($\:\rho\:)$	-0.0081	0.0144	-0.560

Notes: Estimates in bold and italics represent statistically significant value at 99% level, while *estimates in italics*, represent statistically significant value at 95% level. Total sample size = 113,760 (61143 censored and 52617 observed). Final Log-Likelihood = 76992.72.

To further examine complex, potentially nonlinear associations, we applied several ML models, including OLS, Ridge, Lasso, Random Forest, and XGBoost, to the entire dataset. All models were trained using 80% of the data, with performance assessed on a held-out 20% test set (see Table 4). For Ridge regression, the optimal regularisation parameter was α = 10, while for Lasso regression it was α = 0.001. The best Random Forest model was identified with 200 trees, maximum depth of 20, minimum samples per leaf of 2, and minimum samples split of 2. The top-performing XGBoost model used 200 trees, maximum tree depth of 8, a learning rate of 0.2, a subsample ratio of 1, and a feature subsample ratio of 0.8 .

**Table 5:** Model performance comparison (test set).

Model	*R*²	RMSE	MAE
Tobit (Outcome)	0.025	—	—
OLS	0.022	0.01333	0.00520
Ridge	0.022	0.01333	0.00520
Lasso	0.003	0.01346	0.00545
Random Forest	0.288	0.01137	0.00396
XGBoost	0.288	0.01137	0.00398

Note: Total sample size of test dataset = 28,440.

Linear models exhibited low predictive performance (R² ≈ 0.02 for OLS and Ridge, and R² ≈ 0.003 for Lasso), mirroring the modest fit of the Tobit outcome. In sharp contrast, the Random Forest and XGBoost models achieved substantially higher predictive accuracy (R² ≈ 0.29), and correspondingly lower RMSE and MAE values (Table 5). This improvement demonstrates the advantage of ensemble tree-based models in capturing complex, nonlinear, and interactive effects that classical models may overlook.

**Fig. 8:**
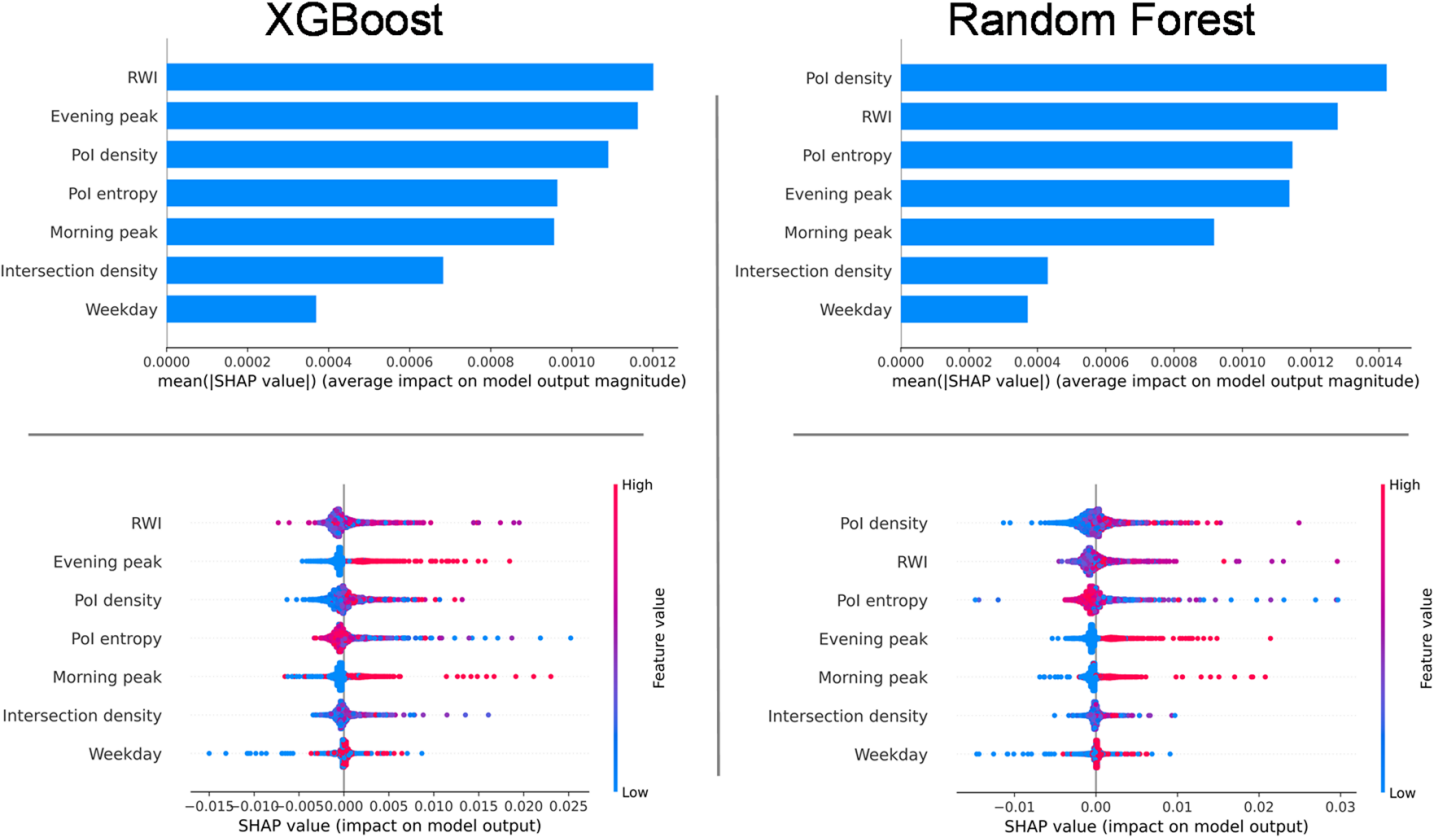
SHAP summary and bar plots for feature importance.

To interpret the determinants of CCI identified by the best-performing machine learning models, we conducted a SHAP (SHapley Additive exPlanations) analysis^45^, which enables robust, model-agnostic explanations of feature importance. The SHAP bar and summary plots for both Random Forest and XGBoost (Fig. 8) provide valuable insights into the relative influence of BE and temporal features.

Across both ensemble models, PoI density consistently emerged as one of the strongest predictors of crowding, reinforcing the conclusion from the Tobit model that stations located in dense, activity-rich environments are most susceptible to contributing to link-level congestion. However, a notable distinction in the XGBoost model is the prominence of Meta’s RWI (a proxy for local economic activity), which was identified as the most influential predictor, followed by evening peak and PoI density. This highlights the capacity of gradient-boosted methods to capture nuanced relationships between economic vibrancy and station-level crowding, an effect that was less apparent in the linear or Tobit frameworks. Random Forest also ranked PoI density and RWI highly, but assigned greater relative importance to PoI entropy, further emphasizing the mitigating role of land-use diversity.

Temporal variables, including evening peak, morning peak, and weekday, consistently contributed to predictions in both models, with evening peak generally exerting a stronger effect than morning peak. Intersection density was also an important driver, albeit to a lesser degree than PoI-related measures and temporal factors. The SHAP summary plots provide additional clarity on the directionality of effects: higher PoI density, RWI, and peak period indicators all increase predicted CCI, while greater PoI entropy (i.e., a more balanced mix of land uses) tends to moderate or reduce crowding severity.

It is important to note some limitations and caveats regarding the interpretation of these results. First, while SHAP values enable comparison of variable importance within a given model, direct comparison of the magnitude of SHAP values across models should be approached with caution, as ensemble methods may capture interactions and non-linearities differently. Second, the ML and SHAP analyses were conducted on the full dataset without distinction between selection and outcome processes, unlike the two-stage structure of the Tobit model. This means that ML feature importance reflects overall predictive relevance, rather than causal mechanisms specific to the likelihood or intensity of crowding. Lastly, although Random Forest and XGBoost outperformed linear models in terms of predictive accuracy, their explanations may be less directly interpretable, especially where complex interactions drive the model outputs.

Despite these caveats, the SHAP analysis strengthens confidence in the robustness of the findings. The consistency in top predictors across methods underscores the dominant roles of land-use intensity, economic activity, and temporal demand surges in driving metro crowding. At the same time, the additional insights from ML and SHAP highlight the value of incorporating flexible, non-linear approaches for diagnosing the spatial and temporal heterogeneity underlying transit congestion.

### Extensions of CCI

The CCI framework is inherently flexible and can be extended to incorporate different objective measures of crowding as weights to the station contributions to link-level flows. The base formulation (see Eq. (4)) averages contributions across different links $\:{L}_{hd}$, where $\:{r}_{slhd}\:\ne\:0$. Building on this structure, we evaluate two extensions of the framework by introducing crowding level sensitive weights, scaling links by how crowded they are compared to its capacity.

First, we weight by absolute excess passengers beyond capacity on each link. The weighted CCI is then given by:

5 $$\:{CCI}_{shd}^{\left(abs\right)}=\frac{1}{\left|{L}_{hd}\right|}\sum\:_{l\in\:{L}_{hd}}{w}_{shd}^{\left(abs\right)}\:{r}_{slhd}$$

Where,

$\:{CCI}_{shd}^{\left(abs\right)}=$ Absolute-excess weighted crowding contribution index of station $\:s$ during hour $\:h$ on day $\:d$.

$\:{w}_{shd}^{\left(abs\right)}=\:{F}_{lhd}-{C}_{lhd}$ ; denoting the weight based on excess passengers beyond capacity on link $\:l$ during hour $\:h$ on day $\:d$.

This extension retains the original contribution shares $\:{r}_{slhd}\:$while giving larger influence to links with more passengers above capacity, i.e., a direct measure of how many riders are causing overload.

Second, we develop an extension of CCI weighted by proportional overload (percentage above capacity). This is given by:

6 $$\:{CCI}_{shd}^{\left(ratio\right)}=\frac{1}{\left|{L}_{hd}\right|}\sum\:_{l\in\:{L}_{hd}}{w}_{shd}^{\left(ratio\right)}\:{r}_{slhd}$$

Where,

$\:{CCI}_{shd}^{\left(ratio\right)}=$ Ratio-excess weighted crowding contribution index of station $\:s$ during hour $\:h$ on day $\:d$.

$\:{w}_{shd}^{\left(ratio\right)}=(\frac{{F}_{lhd}}{{C}_{lhd}}-1)$ ; denoting the weight based on the proportional excess over capacity on link $\:l$ during hour $\:h$ on day $\:d$.

Here, links that are a higher fraction over capacity receive more emphasis, distinguishing cases with similar $\:{r}_{slhd}\:$but different $\:{F}_{lhd}/{C}_{lhd}$.

**Fig. 9:**
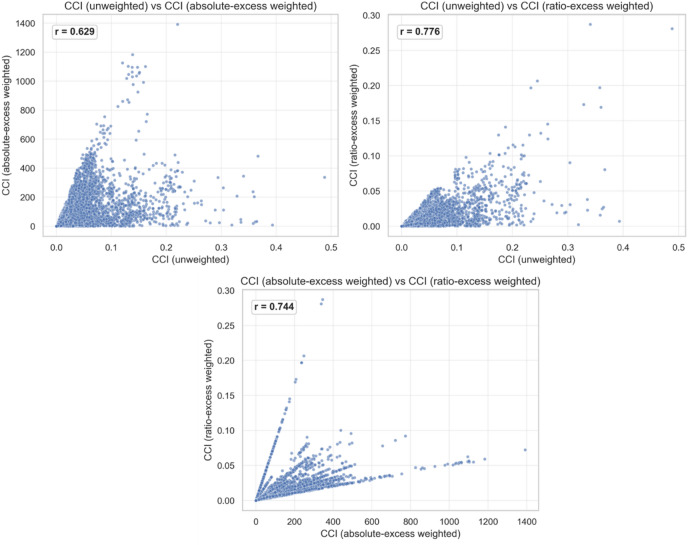
Scatter plots of CCI variants.

We additionally compared the three CCI formulations to assess how weighting changes the index (see Fig. 9). The correlation between CCI (unweighted) and CCI (absolute-excess weighted) is *r* = 0.629, indicating moderate to high similarity. It can be argued that weighting by excess passengers $\:({F}_{lhd}-{C}_{lhd})$ reorders contributions in cases where a modest share $\:{r}_{slhd}\:$coincides with large overloads, elevating stations whose downstream links carry many riders above capacity. In contrast, CCI (unweighted) and CCI (ratio-excess weighted) exhibit *r* = 0.776, reflecting a higher level of alignment. The high correlation emphasizes that proportional overload $\:(\frac{{F}_{lhd}}{{C}_{lhd}}-1)$preserves more of the unweighted structure while still highlighting links that are relatively more crowded (e.g., 200% vs. 117% of capacity). Together, these results demonstrate the flexibility of the CCI framework. The core contribution share $\:{r}_{slhd}\:$is retained, and analysts can tune emphasis toward absolute severity (passengers) or relative severity (percentage) without altering the underlying structure of the index, supporting different operational priorities and planning objectives.

## Discussion

Unlike load-factor or standee-density, which treat each train as a single “black box,” CCI allocates only the overcapacity portion of passenger flow back to the specific destination station that generated it (Sect. 2.5, Eqs. 3–4). This attribution lets agencies answer questions such as “Which station’s alightings caused Link 12 to exceed its 1,200 pax/hr design limit between 8–9 AM?” rather than merely reporting that “Train A was 120% full.” By decomposing crowding at the link and station level, CCI reveals hidden hotspots that would be averaged out by system-wide statistics and provides a direct bridge from in-vehicle conditions back to station-area land uses. In essence, by addressing the fundamental question that “travel is a derived demand of activity,” this study offers novel insights into which aspects of urban structure and land use are driving congestion, thereby highlighting the innovative contribution of the proposed framework.

In the short term, CCI offers several actionable operational insights. Morning peaks at Noida Sector 62 and Noida Sector 52 (Sect. 3.1, Fig. 6) suggest that turning back additional trains just before these termini would absorb excess arrivals, instead of sending every train to the far end of the line. Similarly, CCI can guide dynamic fleet deployment by indicating when higher-capacity trainsets should run on segments feeding high-CCI destinations during peak hours. Persistent off-peak crowding at intermodal hubs such as Shahdara and Shaheed Sthal points to service-quality performance indicators: increasing buffer times or dwell times at these locations can improve schedule reliability and passenger comfort. The fact that New Delhi, Chandni Chowk and Anand Vihar, stations with strong bus, BRT or paratransit connections also exhibit elevated off-peak CCI underscores the value of strengthening feeder services to distribute boarding loads more evenly throughout the day.

Over the longer term, CCI can inform strategic land-use and urban development decisions. The pronounced one-directional flows into eastern Noida corridors during the morning and the central business district interchanges in the evening highlight the need to decentralize activity hubs. Encouraging the development of satellite business districts near key nodes such as the Botanical Garden interchange would reduce peak-direction pressure. Equally, prioritizing mixed-use zoning incentives around high-density, mono-use stations, for example, Noida Electronic City would balance residential, office and retail functions, smoothing arrival patterns and mitigating sudden surges.

In data-constrained contexts typical of many Global South cities, CCI’s reliance on widely available AFC and GTFS feeds offers a low-cost, scalable diagnostic tool for both transport agencies and planning departments. By directly linking crowded links back to station environs, CCI supports targeted operational tweaks and longer-term land-use adjustments even in the absence of AVL systems. Looking forward, the CCI framework can be extended by integrating probabilistic transit assignment to capture route choice variability, incorporating real-time AVL occupancy data to calibrate link-level estimates, augmenting schedules with temporal elasticity measures of demand sensitivity, and coupling with land-use change models to forecast how new developments will shift crowding hotspots. These enhancements would further cement CCI’s role as an actionable, theory-driven metric for sustainable metro operation and urban planning in rapidly growing megacities.

## Conclusions

This study advances the understanding of metro crowding by introducing a CCI to quantify the role of destination stations in contributing to link-level crowding. Unlike conventional crowding measures that focus on station-level or aggregate passenger flows, the CCI provides a granular, link-level perspective, offering valuable insights into how specific stations shape congestion patterns across the metro network. By incorporating spatiotemporal variations in crowding contributions and examining their relationship with BE factors, this study bridges critical gaps in transit research, particularly in the context of rapidly urbanising cities in the Global South. Through this study, an empirical analysis of the Delhi Metro, India’s largest and busiest rapid transit network, AFC data from September 2023, comprising 80,042,580 cleaned trips across 237 metro stations. Given the high ridership densities and infrastructure constraints characteristic of transit systems in the Global South, this study provides a critical framework for understanding and managing metro crowding in similar urban environments.

A major contribution of this study is the development of the CCI, a novel indicator that systematically captures the extent to which passengers alighting at a station contribute to overcapacity crowding on preceding transit links. This framework enables transit agencies to distinguish whether crowding is the result of a few dominant stations or a more widespread phenomenon, thereby guiding targeted interventions such as service modifications, demand management strategies, and land use planning. The study further reveals the strong influence of BE characteristics on station’s contributions to link-level crowding. Our results show that PoI density consistently amplifies a station’s contribution to link-level crowding, while higher PoI entropy mitigates it, underscoring the value of land-use diversity around stations Meta’s RWI and intersection density also emerge as positive drivers of crowding contributions, highlighting how local economic vitality and street connectivity shape travel demand. Notably, both the Tobit regression and our Random Forest/XGBoost models rank these same built-environment variables as the strongest predictors of CCI, demonstrating the robustness of these relationships across econometric and machine-learning frameworks. Together, these findings suggest that planners can prevent localised overcrowding by promoting mixed-use development to diversify station catchments and by tailoring service adjustments, such as targeted short-turns and dynamic fleet deployment to align with the unique land-use and socioeconomic profiles of high-impact stations. Furthermore, two extensions of the CCI framework that incorporate crowding-sensitive weighting factors, based on absolute and proportional overloads were evaluated, demonstrating the adaptability of the proposed index to capture varying congestion intensities across links.

By proposing a novel indicator to measure link-level crowding contributions, this study provides an actionable framework for transit planners and policymakers to identify congestion hotspots and implement data-driven interventions. The findings emphasise that BE characteristics and temporal patterns play a critical role in shaping crowding dynamics, reinforcing the need for integrated land-use and transport planning. However, the study has several limitations that should be considered when interpreting its findings and contributions. Firstly, the non-availability of AVL data necessitated assumptions about the distribution of travel time between stations. While reasonable approximations were made, a more precise understanding of train movements and dwell times would enhance the accuracy of crowding estimations. Secondly, the study relied on a shortest-path assumption for passenger route choice due to the unavailability of detailed individual journey data. In reality, passengers may base their route decisions on factors beyond just minimising travel time, such as train frequency, crowding conditions, and personal preferences. Incorporating empirical route choice data in future research would improve the robustness of the CCI framework. Thirdly, the study focuses on the role of destination stations in contributing to link-level crowding, while origin stations also influence congestion patterns. However, since the CCI framework quantifies how passengers alighting at destination stations affect preceding link loads, the analysis was structured around destination-based contributions. Future research could explore methodologies to incorporate the role of origins in shaping link-level crowding. Fourthly, this study defines overcapacity crowding using a fixed capacity threshold, but policymakers may require a more flexible definition, particularly when identifying extreme overcrowding. Future studies could refine the CCI framework by adjusting the congestion threshold, for instance, considering scenarios where link loads exceed twice the capacity to better reflect varying levels of crowding severity. Fifthly, the analysis was constrained by the availability of limited BE data, meaning that only selected characteristics were included. Other factors, such as station design elements and transfer penalties, could also play a role in determining crowding contributions. Future studies could expand the range of explanatory variables to provide a more comprehensive understanding of what drives crowding at a network level. Finally, this study focuses on a single metro system, the Delhi Metro, during a specific time period. While the findings offer valuable insights, further research is needed to test the generalisability of the proposed framework across different transit systems, especially in cities with varying urban structures and travel behaviours utilising longer periods of data. Exploring how crowding patterns evolve over longer time horizons and under different policy interventions could provide further validation and refinement of the approach. However, the CCI framework is inherently extensible, it can readily incorporate AVL-derived occupancy data, probabilistic route assignment, flexible capacity thresholds, expanded BE variables, and origin-based attributions, enabling future applications to overcome the above limitations and adapt to diverse operational contexts.

## Data Availability

The datasets generated and/or analysed during the current study are not publicly available due to restrictions apply to the availability of the smartcard and QR code data from Delhi Metro Rail Corporation, but are available from the corresponding author on reasonable request.

## References

[1] Varghese, V. & Moniruzzaman Md, Chikaraishi, M. Environmental sustainability or equity in welfare? Analysing passenger flows of a mass rapid transit system with heterogeneous demand. *Res. Transp. Econ.* **97**, 101258 (2023).

[2] Li, Z. & Hensher, D. Crowding in public transport: A review of objective and subjective measures. *J. Public. Trans.* **16** (2), 107–134 (2013).

[3] Choi, J., Lee, Y. J., Kim, T. & Sohn, K. An analysis of metro ridership at the station-to-station level in Seoul. *Transp. (Amst)*. **39** (3), 705–722 (2012).

[4] Zhao, J., Deng, W., Song, Y. & Zhu, Y. Analysis of metro ridership at station level and station-to-station level in nanjing: an approach based on direct demand models. *Transp. (Amst)*. **41** (1), 133–155 (2014).

[5] Pelletier, M. P., Trépanier, M. & Morency, C. Smart card data use in public transit: A literature review. *Transp. Res. Part. C Emerg. Technol.* **19** (4), 557–568 (2011).

[6] Kim, H., Faroqi, M. & Mesbah, J. Applications of transit smart cards beyond a fare collection tool: A literature review. *Adv. Transp. Stud.* **45**, 107–122 (2018).

[7] Kusakabe, T., Iryo, T. & Asakura, Y. Estimation method for railway passengers’ train choice behavior with smart card transaction data. *Transp. (Amst)*. **37** (5), 731–749 (2010).

[8] van Oort, N. & Cats, O. Improving public transport decision making, planning and operations by using Big Data Cases from Sweden and the Netherlands. *2015 IEEE 18th International Conference on Intelligent Transportation Systems, Gran Canaria, Spain.* pp. 19-24 (2015).

[9] Cheriyamadam, P. E. *Estimating Train Passenger Load from Automated Data Systems: Application To London Underground* (Massachusetts Institute of Technology (MIT), 2010).

[10] Zhu, Y., Koutsopoulos, H. N. & Wilson, N. H. M. A probabilistic Passenger-to-Train assignment model based on automated data. *Transp. Res. Part. B: Methodological*. **104**, 522–542 (2017).

[11] Hörcher, D., Graham, D. J. & Anderson, R. J. Crowding cost Estimation with large scale smart card and vehicle location data. *Transp. Res. Part. B: Methodological*. **95**, 105–125 (2017).

[12] Yap, M., Cats, O. & van Arem, B. Crowding valuation in urban Tram and bus transportation based on smart card data. *Transportmetrica A: Transp. Sci.* **16**, 23–42 (2020).

[13] Basso, F. et al. Crowding on public transport using smart card data during the COVID-19 pandemic: new methodology and case study in Chile. *Sustain. Cities Soc.* **96**, 104712 (2023). 37313370 10.1016/j.scs.2023.104712 PMC10249364

[14] Skoufas, A., Cebecauer, M., Burghout, W., Jenelius, E. & Cats, O. Assessing contributions of passenger groups to public transportation crowding. *J. Public. Trans.* **26**, 100110 (2024).

[15] Cervero, R. & Kockelman, K. Travel demand and the 3Ds: Density, diversity, and design. *Transp. Res. D Transp. Environ.* **2** (3), 199–219 (1997).

[16] Siemiatycki, M. Message in a metro: Building urban rail infrastructure and image in Delhi, India. *Int. J. Urban Reg. Res.* **30** (2), 277–292 (2006).

[17] Iseki, H., Liu, C. & Knaap, G. The determinants of travel demand between rail stations: A direct transit demand model using multilevel analysis for the Washington D.C. Metrorail system. *Transp. Res. Part. Policy Pract.* **116**, 635–649 (2018).

[18] Gan, Z., Yang, M., Feng, T. & Timmermans, H. J. P. Examining the relationship between built environment and metro ridership at station-to-station level. *Transp. Res. D Transp. Environ.* **82**, 102332 (2020).

[19] Abdullah, R., Xavier, B. D., Namgung, H., Varghese, V. & Fujiwara, A. Managing transit-oriented development: A comparative analysis of expert groups and multi-criteria decision making methods. *Sustain. Cities Soc.* **115**, 105871 (2024).

[20] Caset, F., Blainey, S., Derudder, B., Boussauw, K. & Witlox, F. Integrating node-place and trip end models to explore drivers of rail ridership in Flanders, Belgium. *J. Transp. Geogr.* **87**, 102796 (2020).

[21] An, D., Tong, X., Liu, K. & Chan, E. H. W. Understanding the impact of built environment on metro ridership using open source in Shanghai. *Cities* **93**, 177–187 (2019).

[22] Li, S. et al. Spatially varying impacts of built environment factors on rail transit ridership at station level: A case study in Guangzhou, China. *J. Transp. Geogr.* **82**, 102631 (2020).

[23] Yang, L., Yu, B., Liang, Y., Lu, Y. & Li, W. Time-varying and non-linear associations between metro ridership and the built environment. *Tunn. Undergr. Space Technol.* **132**, 104931 (2023).

[24] Cui, M. et al. How do access and Spatial dependency shape metro passenger flows. *J. Transp. Geogr.* **123**, 104069 (2025).

[25] Liu, M., Liu, Y. & Ye, Y. Nonlinear effects of built environment features on metro ridership: an integrated exploration with machine learning considering Spatial heterogeneity. *Sustain. Cities Soc.* **95**, 104613 (2023).

[26] Tao, S., Rowe, F. & Shan, H. Unveiling the influence of behavioural, built environment and socio-economic features on the Spatial and Temporal variability of bus use using explainable machine learning. ArXiv Preprint arXiv:240305545 (2024).

[27] Delhi Development Authority. Master Plan Delhi -2041 [Internet]. Delhi, India; 2023 [cited 2024 Dec 28]. Available from: https://dda.gov.in/sites/default/files/inline-files/Draft%20MPD%202041%20%28English%2909062021_compressed_0.pdf

[28] Empino, J. et al. Smart commuting: Exploring machine learning approaches to understanding the metro rail transit system. In: *2023 International Conference on Electronics, Information, and Communication (ICEIC)* pp. 1–4 (IEEE, 2023).

[29] Pieroni, C., Giannotti, M., Alves, B. B. & Arbex, R. Big data for big issues: Revealing travel patterns of low-income population based on smart card data mining in a global south unequal city. *J Transp Geogr [Internet]* Available from: https://www.sciencedirect.com/science/article/pii/S0966692321002568 **96**, 103203 (2021).

[30] World Population Review & Delhi India population 2024 [Internet]. 2024 [cited 2024 Dec 19]. Available from: https://worldpopulationreview.com/cities/india/delh

[31] Chi, G., Fang, H., Chatterjee, S. & Blumenstock, J. E. Microestimates of wealth for all low- and middle-income countries. *Proceedings of the National Academy of Sciences* **119** (3), (2022). 10.1073/pnas.2113658119 PMC8784134 35017299

[32] Lizana, M., Choudhury, C. & Watling, D. Using smart card data to model public transport user profiles in light of the COVID-19 pandemic. *Travel Behav Soc [Internet]* **33**, 100620 (2023). Available from: https://www.sciencedirect.com/science/article/pii/S2214367X23000716

[33] Medina, S. A. O. Inferring weekly primary activity patterns using public transport smart card data and a household travel survey. *Travel Behav. Soc.* **12**, 93–101 (2018).

[34] Ann, S., Jiang, M., Mothafer, G. I. & Yamamoto, T. Examination on the influence area of Transit-Oriented development: considering multimodal accessibility in new Delhi, India. *Sustainability* **11** (9), 2621 (2019).

[35] Chen, E., Ye, Z., Wang, C. & Zhang, W. Discovering the spatio-temporal impacts of built environment on metro ridership using smart card data. *Cities* **95**, 102359 (2019).

[36] Singh, Y. J., Fard, P., Zuidgeest, M., Brussel, M. & van Maarseveen, M. Measuring transit oriented development: a Spatial multi criteria assessment approach for the City region Arnhem and Nijmegen. *J. Transp. Geogr.* **35**, 130–143 (2014).

[37] Csardi, G. & Nepusz, T. The Igraph software package for complex network research. *InterJournal Complex. Syst.* **1695,** 1-9 (2006).

[38] Delhi Metro Rail Corporation. Annual Report 2023-24 [Internet]. 2024 [cited 2024 Nov 6]. Available from: https://backend.delhimetrorail.com/documents/7611/Annual-Report-2023-24-English.pdf

[39] Amemiya, T. Tobit models: A survey. *J. Econom*. **24**, 3–61 (1984).

[40] Hastie, T., Tibshirani, R., Friedman, J. H. & Friedman, J. H. *The Elements of Statistical Learning: Data mining, inference, and Prediction* Vol. 2 (Springer, 2009).

[41] Breiman, L. Random forests. *Mach. Learn.* **45** (1), 5–32 (2001).

[42] Chen, T., Guestrin, C. & Xgboost A scalable tree boosting system. In: *Proceedings of the 22nd acm sigkdd international conference on knowledge discovery and data mining* pp. 785–94 (2016).

[43] Ding, C., Cao, X. J. & Næss, P. Applying gradient boosting decision trees to examine non-linear effects of the built environment on driving distance in Oslo. *Transp. Res. Part. Policy Pract.* **110**, 107–117 (2018).

[44] Wang, Z., Liu, S., Lian, H. & Chen, X. Investigating the nonlinear effect of land use and built environment on public transportation choice using a machine learning approach. *Land* **13** (8), 2024 (2012).

[45] Lundberg, S. M. & Lee, S. I. A unified approach to interpreting model predictions. *Adv. Neural Inf. Process. Syst.* **30**, (2017).

